# Secondary Succession under invasive species (*Pteridium aquilinum*) conditions in a seasonal dry tropical forest in southeastern Mexico

**DOI:** 10.7717/peerj.6974

**Published:** 2019-05-28

**Authors:** Alberto Jean Baptiste, Pedro A. Macario, Gerald A. Islebe, Benedicto Vargas-Larreta, Luciano Pool, Mirna Valdez-Hernández, Jorge O. López-Martínez

**Affiliations:** 1 Departamento de Agricultura, Sociedad y Ambiente, El Colegio de la Frontera Sur, Chetumal, Quintana Roo, Mexico; 2 Departamento de Conservación de la Biodiversidad, Herbarium, El Colegio de la Frontera Sur, Chetumal, Quintana Roo, Mexico; 3 División de Estudios de Postgrado e Investigación, Instituto Tecnológico de El Salto, El Salto, Durango, Mexico; 4 Departamento de Agricultura, Sociedad y Ambiente, El Colegio de la Frontera Sur, Campeche, Campeche, Mexico; 5 Cátedras, CONACYT, Ciudad de México, Mexico

**Keywords:** Yucatán Peninsula, Secondary succession, Bracken fern, Fire, Invasive species

## Abstract

The role of invasive species in ecosystem functioning represents one of the main challenges in ecology. *Pteridium aquilinum* is a successful cosmopolitan invasive species with negative effects on the ecological mechanisms that allow secondary succession. In this study, we evaluated the influence of *P. aquilinum*on secondary succession under different disturbances in a seasonal dry forest of the Yucatán Peninsula. We determined species richness, composition and the relative importance value in four sampling units. Fabaceae followed by Asteraceae, Meliaceae, Rubiaceae, Sapindaceae and Verbenaceae were the most species rich families. A dissimilarity analysis determined significant differences in beta diversity between sampling units. With a generalized linear model we found that species richness was best explained by site conditions, followed by calcium and soil organic matter. Also, the generalized linear model showed that abundance resulted in a strong correlation with site conditions and soil characteristics. Specific soil conditions related to phosphoro and calcium were also detected as beneficiary to the successional processes. Our results suggest that applying fire restriction and periodic cutting of the bracken fern, this can increase a higher diversity of species.

## Introduction

*Pteridium aquilinum* (L.) Kuhn is considered one of the most successful global invasive species. Its distribution is related to processes of land use change derived from human activities, e.g., agricultural and livestock activities (Rymer, 1976). This species is particularly successful when light is not a limiting resource (MacDougall & Turkington, 2005; Schneider, 2006). *P. aquilinum* allelopathic characteristics and their tolerance to a wide range of environmental and soil conditions have helped to colonize almost all terrestrial ecosystems, except for deserts (Gliessman, 1976; Taylor, 1989). They have an underground rhizome which branches into long and short buds, can grow up to 2 m in a single season, which gives it a great capacity for colonization (Fletcher & Kirkwood, 1979). Additionally, through the creation of a physical barrier generated by the density of its canopy, it hinders the establishment of native species (Olson & Wallander, 2002). Similarly, it has been observed that the accumulation of biomass from the dry fronds modifies the frequency and intensity of fires, due to the increase of available fuel (Frankland, 1976; Crane, 1990). This exhausts the seed bank and limits the growth of seedlings (Da Silva Matos SR da de, 2006). The lack of competitors for limiting resources (Vandermeer, 1972), the absence of pests and herbivory, and their resistance to fire due to their ability to regrow from their rhizomes, give them strategic advantages over native species (Tolhurst & Turvey, 1992). Studies from Brazil, Ecuador, the Dominican Republic and Rwanda found that former active agricultural regions are currently held back due to the interruption of secondary succession processes (Hartig & Beck, 2003; Gallegos et al., 2015). In Mexico, most of the studies have been carried out in the south, mainly in the states of Quintana Roo, Campeche and Oaxaca (Schneider, 2006; Suazo-Ortuño et al., 2014). Likewise, it has been observed that the lack of strategies for their control can generate a substantial growth in a relatively short time, e.g., Schneider & Fernando (2010) found in the Calakmul reserve, that their coverage increased from 40 km^2^ to 80 km^2^ between 1982 and 2010. It was also observed that landscapes with invaded areas are less productive, have a reduced biological diversity and show severe impacts in the socio-economic dynamics of the affected regions, because the areas cannot be used by the owners (Schneider, 2004; Schneider, 2006; Suazo-Ortuño et al., 2014). Currently, insufficient studies have been developed in seasonally dry tropical forests that provide information on the historical effect and management of areas invaded by *P. aquilinum*, and its impact on the richness and floristic composition, and their influence on succession. Strategies are needed to restore areas affected by one of the most important invasive species in the world. It is important to mention that the establishment of invasive species affects the possibility of maintaining the net primary production of the ecosystems (Ewel & Putz, 2004; Gibbons et al., 2017; Feng, Fouqueray & Van Kleunen, 2019). Consequently, the aim of this work was to determine the effect of the invasion of *P. aquilinum* on secondary succession in a seasonally dry tropical forest.

## Materials & Methods

### Study site

**Figure 1:**
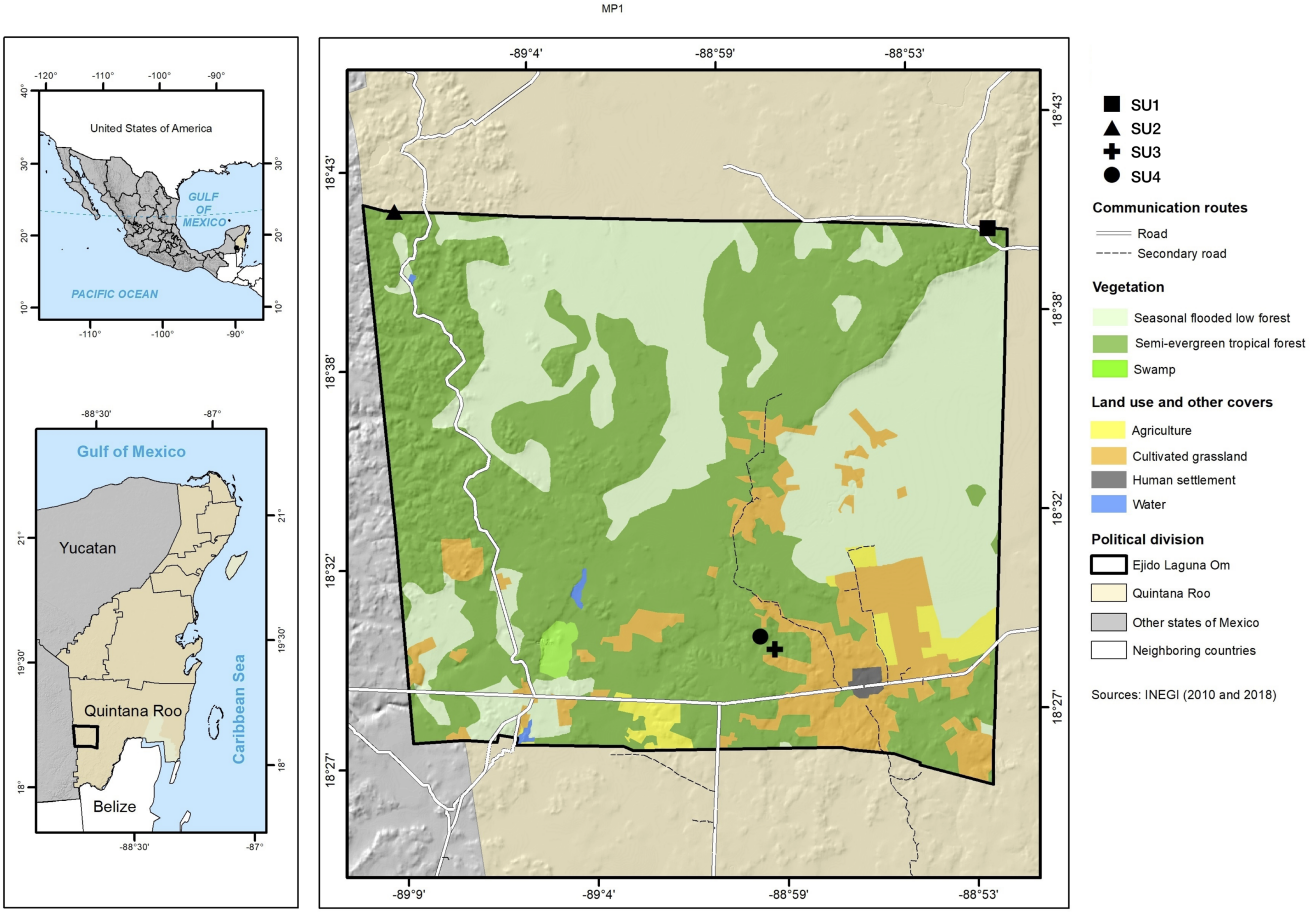
Study site. Location of the four sampled sites and information on vegetation types and cover use.

The study was carried out in the *ejido* (communally managed land) Laguna Om (−89.15W 18.70N, −88.87W 18.40N WGS 84) in the south of the state of Quintana Roo, Mexico with an area of 84,998 ha (Fig. 1). The *ejido* is located in the geological formation called *Petén*, which belongs to the Paleocene-Eocene and is characterized by massive, compact macro and microcrystalline limestones, which have yellow to white coloring, with brown parts stained by iron oxides (Wright, 1967; INEGI, 1993). The terrain is flat with an undulated microrelief, and with wide depressions that present small plains. Its altitude above sea level varies between 100 and 150 m (García, 1997; INEGI, 1993; IUSS Working Group WRB, 2015). The climate is Aw (x’) i, warm subhumid, with precipitation in summer and some in the winter (García, 1997). The climatological station located in the center-south part of the *ejido* reports 1,290 mm/year of precipitation and an average annual temperature of 26 °C. Soils are Leptosols, Vertisols and Gleysols (INEGI, 1993). The dominant vegetation in the study area is seasonal dry tropical forest, and the terrain is low seasonal flooded forest and savanna (Miranda & Hernández, 1963).

### Sampling design

Sampling units were selected based on several conditions. Each of the four sampling units has eight plots (SU) of 10 ×10 m (100 m^2^), which were randomly selected. This gives a total of 32 plots. To evaluate the importance of the soil properties on the richness and abundance of species, soil samples were taken in all the sampling units. 12 soil samples per plot were mixed, giving one composite sample of 2 kg for each sampling unit. Soil analyses were based on the Official Mexican Standard (NOM) 021 RECNAT-2000. The soil samples were dried at room temperature (22–25 °C), macerated and sieved with a 10 mm mesh screen and the following characteristics were analyzed: Total nitrogen (Nt, Micro Kjeldahl method), Phosphorus (Olsen method), Potassium (K, AS-12 with ammonium acetate), pH (Walkley and Black method), texture (Bouyoucos, method), Ca, Na, and soil organic matter (SOM).

### Plots characteristics

Three sampling units were established in areas invaded by *P. aquilinum* for approximately 50 years (from 1960 till present). The age and the frequency of fires of the sample units were defined according to the information provided by local farmers. Sampling unit 1 (SU1) consisted of areas with regular periods of burning every two or three years during the last fifty years. The last recorded fire occurred in 2015; vegetation sampling was carried out one year after the last fire (2016). Sampling unit 2 (SU2) was established in areas invaded by ferns during the last 50 years, but the last burning occurred 12 years ago (2007). In SU1 and SU2, fires were natural and the presence of abundant biomass of *P. aquilinum* and together with higher temperatures during the dry season increased fire probability. Contrary, sampling unit 3 (SU3) was established in areas with a similar land use history, but since 2010 fire was controlled and the removal method applied by Macario (2010) was applied. This method consists of weekly cuttings (locally known as *chapeos*) during two months, followed by monthly cuttings throughout the year. Later quarterly and semi-annual cuttings are applied depending on the density of the fern. A fourth sampling unit (SU4) was established in areas that were burned for the last time in 2007 for the establishment of the milpa system (slash-and-burn agriculture), and was left for recovery since 2009. This area never presented invasion by ferns. All the sampling units presented similar conditions in relation to climate, stoniness, slope and altitude.

### Sampling

The sampling was carried out between May and June 2016. In each of the plots the following variables were recorded: diameter at breast height (DBH) of all tree species >0.5 cm and the taxonomic identity of each species (Wyatt-Smith & Foenander, 1962). All registered individuals were identified from dichotomous botanical keys (Standley & Steyermark, 1958) and existing floristic listings (Sousa & Cabrera, 1983; Pennington & Sarukhán, 2005). The community of plant species was characterized in each of the four SUs establishing richness and abundance. The Importance Value Index (IVI) was determined in each of the treatments.

\begin{eqnarray*}IVI=\text{relative density}+\text{relative basal area}+\text{relative frequency} \end{eqnarray*}

Where: relative density = (number of individuals of species/total number of individuals)*100; relative basal area = (basal area of a species/basal area of all species)*100; relative frequency = (frequency of a species/ frequency of all species)*100 (Kent, 2011).

### Data analysis

To compare the taxonomic diversity of the treatments, a rarefaction analysis (interpolation) and extrapolation (prediction) of the Hill numbers were performed, based on sample size and coverage, which represents a unified criterion to contrast the diversity of multiple assemblages (Hsieh, Ma & Chao, 2016). The analysis was carried out based on the order q (richness of species), and richness estimators were determined with the iNEXT software package R (Chambers, 2008; Hsieh, Ma & Chao, 2016). The variation in the composition of species was analyzed through a non-metric multidimensional scaling (NMDS). To reduce the effect of very abundant species, and to level the effect of rare species, the fourth root transformation in the PRIMER-E 6.1.12 software was applied. Statistically significant differences in the composition of tree species between the different plots were calculated with a dissimilarity analysis (ANOSIM) in PRIMER-E 6.1.12 (Clarke & Gorley, 2006). Finally, a generalized linear model (GLM) was carried out between the species richness and abundance and the soil variables with the package glm2 (Marschner, 2011) in the R software (R Development Core Team, 2012). Prior to the analysis, multicollinearity of variables was established to avoid redundancy (Zuur, Hilbe & Ieno, 2013).

## Results

### Composition and floristic richness

There were 2,162 individuals belonging to 33 families, 56 genera and 63 species, recorded in the 32 sampled plots (SU1 to SU4). Of the total recorded individuals, 1884 (87%) were trees and 278 (13%) shrubs. The sampling unit with the highest diversity was SU4 with 32 families, 49 genera, 54 species and 1091 individuals, of which 882 were trees and 209 shrubs. On the other hand, SU1 showed the lowest species richness with 11 species, belonging to nine families, and a total of 193 trees without the presence of shrubs (Table 1). The families with a higher richness of recorded species were Fabaceae with 11 species, followed by Asteraceae, Meliaceae, Rubiaceae, Sapindaceae and Verbenaceae with three species each, contributing 41% of the total of the species registered in the plots. The genera that presented the highest number of species were *Lonchocarpus* with five, and *Thevetia*, *Eugenia* and *Coccoloba* with two species respectively. The largest number of individuals was distributed among the following families: Rubiaceae with 572, Araliaceae (237), Fabaceae (233), Polygonaceae (159), Cecropiaceae (134), Asteraceae (115), Ulmaceae (110) and Sapindaceae with 104. Species with higher densities were *Guettarda combsii* Urb. (522), *Dendropanax arboreus* (L.) Decne. & Planch. (237), *Coccoloba spicata* Lundell (158), *Cecropia peltata* L. (134) and *Trema micrantha* (L.) Blume (108), and together formed 54% of the inventoried species.

**Table 1:** Sampling units characteristics with richness of taxa, abundances of shrubs, trees and *Pteridium*. All abundances were calculated with areas of 100 m^2^. Fire frequency is expressed as decadal events for the last 50 years.

**SU**	**Family**	**Genus**	**Species**	**Shrubs abundances**	**Trees abundances**	**Total abundances**	**Fronds of*P. aquilinum***	**Decadal fire frequency**
SU1	9	10	11	0	193	193	3,500–4,000	15
SU2	13	18	20	13	57	70	800–1,100	10
SU3	20	31	34	56	752	808	0	10
SU4	32	49	54	209	882	1,091	0	12
Total	33	56	63	278	1,884	2,162		

**Figure 2:**
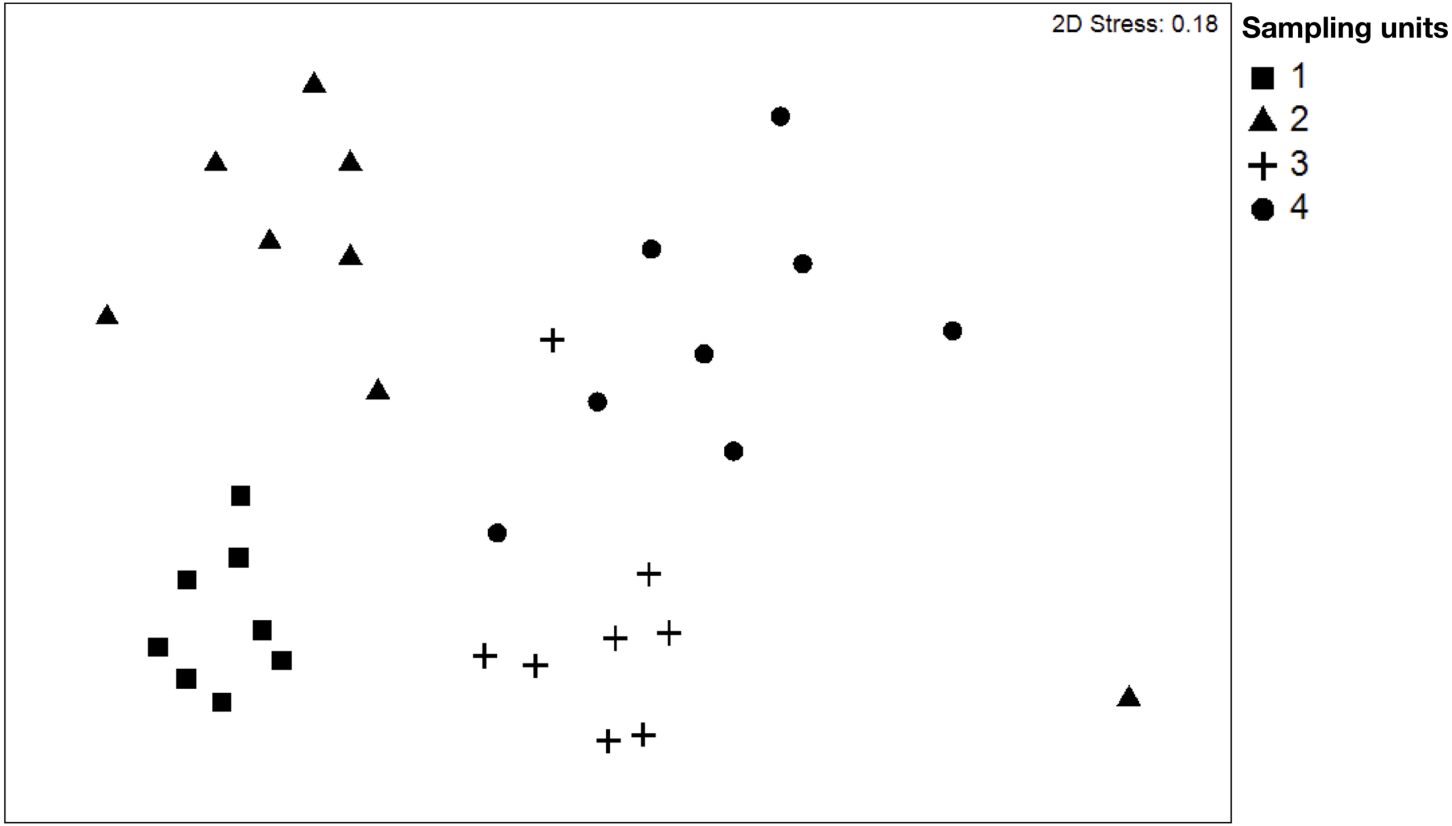
Non-metric multidimensional scaling (NMDS) plot of tree species of the four sampling units.

NMDS (Fig. 2) showed clear differences between sampling units. The stress obtained in the test (0.18%) is probably influenced by the variability in the composition of species of SU4. The dissimilarity of species is a good measure to identify the distribution of the ecological niche between the species. The dissimilarity analysis (ANOSIM) determined significant differences in the dissimilarity between the different plots, indicating that beta diversity varied between sampling units. In this case, the variation percentage ranged from 60% to 91% between the plots. Our results showed that the plots SU1 and SU3 are the plots with a higher degree of differentiation in the composition of species, and SU1 and SU2 are those with a lower percentage of differentiation (Table 2).

**Table 2:** Results of ANOSIM comparisons of community composition among SU, showing test statistics for global and pair-wise comparisons.

	***r***	**p**
All SU	0.0714	0.01
SU1, SU2	0.68	0.02
SU1, SU3	0.917	0.02
SU1, SU4	0.845	0.02
SU2, SU3	0.635	0.05
SU2, SU4	0.607	0.02
SU3, SU4	0.652	0.03

Statistically significant differences were observed for species richness among the evaluated sampling units. It was observed that species richness decreased from SU4 to SU1. Likewise, abundance showed the same pattern, except for SU2 which presented the lowest abundance. It was also observed that the sampling effort performed is representative for the richness and composition of the biota of the region (between 98 and 99% for SU1, SU3 and SU4 and 88% for SU2; Table 3).

**Table 3:** Observed and estimated species richness for all woody plants >0.5 cm dbh. Different letters indicate significant differences. Raref., rarefaction (for 70 individuals); S.obs. observed; dD., estimated (with 70 individuals), qD.LCL, qD.UCL the bootstrap lower and upper confidence limits for the diversity of order q at the specified level in the setting (with a default value of 0.95); SC.LCL, SC.UCL = the bootstrap lower and upper confidence limits for the expected sample coverage at the specified level in the setting (with a default value of 0.95).

**SM**	**M**	**N**	**S.obs**	**qD.LCL, qD.UCL**	**qD**	**SC.LCL, SC.UCL**	**SC**
SU1	8	193	11a	9.40–12.62	9.4a	8.40–10.32	0.99
SU2	8	70	20b	16.4–25.86	18.78bc	15.53–22.03	0.88
SU3	8	808	34c	29.63–38.37	15.21c	14.28–16.19	0.99
SU4	8	1,091	54d	47.47–60.53	22.41db	21.45–23.38	0.98
Total	32	2,162	63				

### Relative Importance value index (IVI)

**Figure 3:**
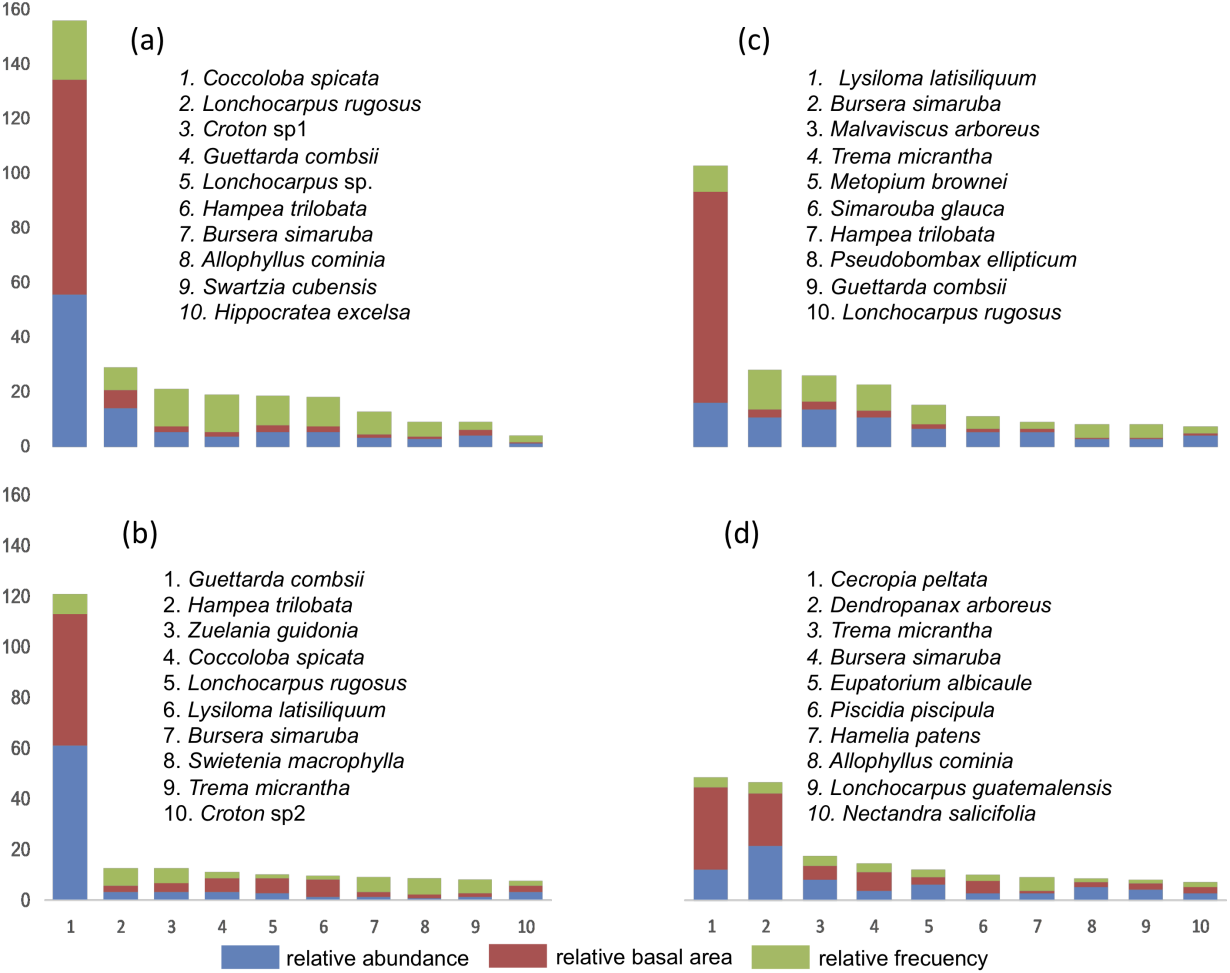
List of tree species with 90% of the Relative Importance Value Index (IVI) in each plot. (A) SU1, (B) SU2, (C) SU3, (D) SU4.

The IVI was obtained in each of the four SU and IVI data for each of the species are presented (Fig. 3). In SU1, the species with the highest IVI are *Coccoloba spicata, Lonchocarpus rugosus* Benth., *Croton* sp1, *Hippocratea excelsa* Kunth. and *Diospyros verae-crucis* Standley. Regarding SU2, the species that characterize this community considering the highest IVI were: *Lysiloma latisiliquum* (L.) Benth., *Bursera simaruba* (L) Sarg., *Malvaviscus arboreus* Cav., *Trema micrantha, Metopium brownei* (Jacq.) Urb., *Simarouba glauca* DC and *Guettarda combsii*. The characteristic species of SU3 based on IVI were *Guettarda combsii, Hampea trilobata* Stand., *Zuelania guidonia* (Sw.) Britton & Millsp., *Coccoloba spicata, Lonchocarpus rugosus* Benth., *Lysiloma latisiliquum*, *Bursera simaruba*, *Swietenia macrophylla* King, *Trema micrantha* and *Croton* sp2. Finally, the species with greater relative importance in SU4 were *Cecropia peltata*, *Dendropanax arboreus*, *Trema micrantha*, *Bursera simaruba*, *Eupatorium albicaule* Sch. Bip. ex Klatt, *Piscidia piscipula* (L.) Sarg., *Hamelia patens* Jacq., *Allophylus cominia* (L.) Sw., *Lonchocarpus guatemalensis* Benth*., Nectandra salicifolia* Kunth., *Coccoloba spicata* Lundell, *Aegiphila monstrosa* Moldenke, *Bourreria oxyphylla* Stand., *Verbesina gigantea* Jacq. and *Guettarda combsii*.

### Diversity and abundance

The species richness and conditions of the sampling units considering the disturbance frequency resulted in significant differences. SU3 showed almost twice the number of species found in SU1 and SU2. As expected, SU4 was the site with the highest species richness (Table 3). Table 4 summarizes soil characteristics. Soil organic matter and N were excluded from the analysis, as those values indicated collinearity >10. GLM showed a strong correlation between the species richness of tree species. Variables, which explained best species richness, were site conditions, followed by Ca and soil organic matter. Also, GLM showed that abundance resulted in a strong correlation with site conditions and soil characteristics. Species abundance was best explained by site conditions, P, Ca and organic C (Table 5).

**Table 4:** Soil nutrients of SU1, SU2, SU3 and SU4: Nitrogen (N %), Phosphorus (P mg*kg), Potassium (mg*kg), Sodium (Na mg*kg), Calcium (Ca %), soil organic matter (SOM %), C _org_ and pH. ± standard error.

SU	N	SOM	P	K	Na	Ca	C_org_	pH
SU1	0.32 ± (0.04)	4.66 ± (0.6)	0.07 ± (0.02)	2.92 ± (0.5)	0.18 ± (0.02)	2.93 ± (0.6)	5.68 ± (0.27)	7.57 ± (0.06)
SU2	0.25 ± (0.04)	3.2 ± (0.5)	0.05 ± (0.01)	1.95 ± (0.69)	0.13 ± (0.01)	3.82 ± (0.49)	5.23 ± (0.28	7.73 ± (0.03)
SU3	0.19 ± (0.05)	2.61 ± (0.61)	0.03 ± (0.01)	2.9 ± (0.53)	0.12 ± (0.03)	3.4 ± (0.9)	5.85 ± (0.55)	7.71 ± (0.09)
SU4	0.24 ± (0.09)	3.27 ± (1.19)	0.05 ± (0.02)	3.51 ± (1.22)	0.1 ± (0.03)	1.7 ± (0.18)	4.94 ± (0.49)	7.60 ± (0.03)

**Table 5:** Variation partitioning of woody species richness and abundance through General Lineal Model (GLM) and influence of each variable in the model.

**Variable**	**% Total explain model**	****	**Estimate**	**Sta. Error**	***z* value**	***p***
Richness	72.1	(Intercept)	1.77947	1.95533	0.91	0.362789
		SU	0.73231	0.02402	30.49	***0.0001***
		P	−0.49618	1.00638	−0.493	0.621985
		K	0.01219	0.02337	0.521	0.602098
		Na	−0.90082	0.71579	−1.258	0.208212
		Ca	0.12279	0.026	4.723	***2.32E–06***
		C_org_	−0.07272	0.02142	−3.395	***0.000686***
		pH	0.05628	0.24927	0.226	0.821389
Abundance	60.2	(Intercept)	6.54206	5.07398	1.289	0.19728
		SU	0.47363	0.05821	8.136	4.08E-16
		P	6.74966	2.76212	2.444	***0.01454***
		K	0.05896	0.05884	1.002	0.31634
		Na	−4.72098	2.04024	−2.314	***0.02067***
		Ca	0.18468	0.0689	2.681	***0.00735***
		C_org_	−0.14126	0.05598	−2.524	***0.01162***
		pH	−0.69346	0.65187	−1.064	0.28742

## Discussion

The floristic composition in the all evaluated sampling units (SU1 to SU4) was represented mainly by species of the Fabaceae. This agrees with results of secondary vegetation studies carried out in the Yucatán Peninsula (Mizrahi, Ramos-Prado & Jiménez-Osornio, 1997; Pool-Estrella, 1998; Góngora-Chín, 1999; González-Iturbe, Olmsted & Tun-Dzul, 2002; Ceccon, Sánchez & Campo, 2004; Islebe et al., 2015). Other authors like Sánchez-Sánchez & Islebe (2002) and Duno et al. (2012) found that Fabaceae are particularly diverse in the southern region of the Yucatán Peninsula due to the precipitation gradient and its biogeographical affinity to the biota of Central America. Furthermore, it has been widely reported that it is the dominant family in secondary vegetation at different successional stages (Sánchez & Islebe, 1999; Flores & Bautista, 2012; López-Martínez et al., 2013; Valdez-Hernández et al., 2015).

The species with the highest densities recorded in the sampling units is a group of species with resprouting capacities: *C. spicata, L. rugosus, Croton* sp1*, H. trilobata, B. simaruba, G. combsii, Croton* sp2*,* (SU1 and SU2) characteristic of degraded areas. Sites SU3 (fern with cutting removal) and SU4 (bracken free) presented the highest IVI values of species with seed reproduction (*L. latisiliquum, M. brownei, P. ellipticum, C. peltata, N. salicifolia*). Other studies in the Yucatán Peninsula found similar distribution patterns of those tree species in secondary vegetation of seasonal dry forests (Dupuy et al., 2012; Valdez-Hernández et al., 2014; Valdez-Hernández et al., 2019). Differential successional routes can be observed depending on the origin of the disturbance (Ferguson et al., 2003), which explains the dissimilarity in species composition between SU1-3 and SU4. The low values of the IVI in most of the recorded species indicate specific traits like fast growth and differential adaptation to soil and site characteristics. Similar conditions were observed in a seasonal dry tropical forest of northern Quintana Roo where, in hurricane-damaged areas, few species dominated (Sánchez & Islebe, 1999).

The variation of species richness and abundance recorded by the rarefaction results is attributed to the invasion of *P. aquilinum* and the history of land use in the area. This reflects dominance patterns of species, and therefore beta diversity (López-Martínez et al., 2013). The results suggest that while controlling the frequency of fires does not eradicate the invasive species, it can increase the diversity of species, e.g., SU2 showed higher richness than SU1. Also, the low species richness can be explained by the different edaphic conditions present in each sampling unit (depth, stoniness among others). Fires, occasional or frequent, favor the spread of bracken fern. The establishment of species in SU1 and SU2 is limited by shallow soils with little organic matter and characterized by species of secondary forest patches (Suazo-Ortuño et al., 2014; Valdez-Hernández et al., 2014).

Three of the sampling units with *Pteridium* indicate that the recent history of land use contributes to an understanding of the variation of species richness. It has been observed that fires caused in areas dominated by *P. aquilinum* tend to be more intense than those that occur in other types of cover (Cawson et al., 2018). The high frequency of fires has a negative consequence, and greater impact on tree species than on invasive species such as *Pteridium* (Roos et al., 2010). The highest dissimilarity was found between SU1 and SU3, as a consequence of the cutting technique of *P. aquilinum*. This favored environmental heterogeneity and the establishment of short-lived species, as well as species of late successional ages.

Heterogenous environmental conditions of the area explain why the age of the vegetation could influence the results and restricts the regeneration in SU1. Probably, the absence of seed banks and the frequency of burning will eventually interrupt the succession. There was a high floristic dissimilarity among all sampling units (Table 3). The high dissimilarities suggest that most species of the sampling units have a high turnover rate. López-Martínez et al. (2013) reported that significant differences in the dissimilarity between successional age classes indicate that the composition of the species changes during the succession. From other studies analyzing succession in seasonal dry tropical forests in the Yucatán Peninsula, we observe a similar trend (Valdez-Hernández et al., 2014).

Our results show that there is a strong relationship between species richness and abundance with the conditions of the site and some chemical soil variables. In particular, our results showed a direct relationship between species richness and abundance with P and Ca. The positive relationship with P and Ca suggests that concentrations of both elements are available given the dynamics of the organic matter in SU3 and SU4, which has not been interrupted by fire disturbances. Similar soil conditions are reported by Bautista et al. (2005). The relationship between Na and C_org_ suggest that metabolic activity of soil microorganisms (Ceccon, Huante & Campo, 2003; Eaton & Lawrence, 2006; Lange et al., 2015; Rodrigues et al., 2016) are favoring the regeneration of SU3 and SU4. On the other hand, studies carried out in degraded sites found that the concentrations of C_org_ and Na can be reduced for several years before the restoration of soil dynamics begins (Schlesinger & Bernhardt, 2013), which explains low species diversity and abundance in SU1 and SU2.

## Conclusions

Sampling sites with higher fire frequency had lower diversity values and observed species numbers. In sites without fires and bracken removal, no increase in species recruitment was observed. We observed that the control of fern cover with constant cuttings favors richness and species composition, and steps up secondary succession. Specific soil conditions related to P and Ca were also detected as beneficiary to the successional processes.

##  Supplemental Information

10.7717/peerj.6974/supp-1 Supplemental Information 1 Raw data Raw data of species richness, abundances, and samples soils of each plot sampled.

## Additional Information and Declarations

### Data Availability

The following information was supplied regarding data availability: López-Martínez, J.O. (2019). Raw data of diversity, abundance and soil samples (Version Final version) [Data set]. PeerJ. Zenodo. http://doi.org/10.5281/zenodo.2649736.
